# Oral amino acid tracer delivery detects feeding and exercise changes in myofibrillar protein synthesis rates in male adults

**DOI:** 10.14814/phy2.70776

**Published:** 2026-03-03

**Authors:** Michael Mazzulla, Nathan Hodson, Paula J. Scaife, Kenneth Smith, Philip J. Atherton, Nicholas A. Burd, Dinesh A. Kumbhare, Daniel R. Moore

**Affiliations:** ^1^ Faculty of Kinesiology and Physical Education, Department of Exercise Sciences University of Toronto Toronto Ontario Canada; ^2^ MRC‐Versus Arthritis Centre for Musculoskeletal Ageing Research and NIHR Nottingham BRC, Centre of Metabolism, Ageing and Physiology, School of Medicine University of Nottingham Derby UK; ^3^ Department of Health and Kinesiology University of Illinois Urbana‐Champaign Urbana Illinois USA; ^4^ Department of Medicine University of Toronto Toronto Ontario Canada

**Keywords:** amino acids, muscle protein synthesis, resistance exercise, stable isotopes

## Abstract

The efficacy of oral administration of leucine and phenylalanine tracers to measure MyoPS (LEUMyoPS and PheMyoPS, respectively) in response to varying anabolic stimuli was investigated. Participants were randomized to a rested‐fasted (FAST), rested‐fed (FED), or exercise‐fed (EXFED) condition. FED and EXFED consumed a mixed carbohydrate and AA beverage enriched with L‐[1‐^13^C]leucine (25%) and L‐[*ring*‐^2^H_5_]phenylalanine (30%) at rest or after a bout of resistance exercise, while FAST consumed only the equivalent tracer dose. Blood samples were obtained every 30 min for 300 min to determine tracer precursor enrichment with muscle biopsies obtained before and at 120 and 300 min after tracer ingestion to determine MyoPS by the precursor‐product method. LEUMyoPS over 300 min was greater (*p* < 0.01) in EXFED (0.090 ± 0.024%/h; mean ± SD) and FED (0.067 ± 0.028%/h) compared to FAST (0.024 ± 0.015%/h). PHEMyoPS over 300 min was greater (*p* < 0.01) in EXFED (0.128 ± 0.034%/h) and FED (0.098 ± 0.020%/h) compared to FAST (0.056 ± 0.012%/h). There was a positive correlation (*r* = 0.81, *p* < 0.0001) between LEUMyoPS and. Oral essential amino acid tracer ingestion can be used as an alternative method to detect changes in MyoPS in response to mixed macronutrient feeding and feeding plus exercise and may be an option for the study of human muscle protein synthesis where intravenous isotope administration is logistically difficult or prohibitively expensive.

## Graphical abstract

Male participants were randomly assigned to consume [^13^C]leucine and [D5]phenylalanine tracers alone (FAST) or with a complete mixture of amino acids (0.25 g/kg) and carbohydrate (0.75 g/kg) at rest (FED) or after a bout of resistance exercise (EXFED). Plasma enrichment of both tracers increased rapidly after ingestion and remained elevated for up to 4–5 h. Muscle biopsies detected incorporation into myofibrillar proteins, which resulted in expected physiological differences in myofibrillar protein synthetic rates between conditions (EXFED > FED > FAST) regardless of tracer. Our study demonstrates that oral essential amino acid tracer ingestion can be used as an alternative method to traditional intravenous infusion to detect changes in myofibrillar protein synthesis rates in humans.

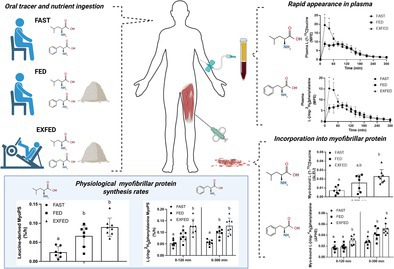

## 1 INTRODUCTION

Skeletal muscle proteins, such as the force‐generating myofibrillar fraction, are in a state of dynamic homeostasis (i.e., “turnover”) whereby they are constantly broken down into their constituent amino acids that can subsequently be utilized to synthesize new proteins. The synthesis of new muscle proteins can be estimated through the use of stable isotope amino acid tracers, which traditionally are provided intravenously as a primed‐constant infusion (Rennie et al., 1994) or as intravenous bolus “flooding” (Garlick et al., 1989) or “pulse” doses (Zhang et al., 2002). Provided labeling of the precursor pool for protein synthesis can be estimated over time, the fractional synthesis of specific muscle proteins can be determined. This is commonly achieved by frequent blood sampling in conditions of nonsteady state isotope enrichment to provide a surrogate for the true precursor tRNA enrichment (Gwin et al., 2021; Smith et al., 1992; van Vliet et al., 2019; Wall et al., 2016; Witard et al., 2014), which is technically difficult to measure given its small pool size and requirement for (repeated) larger muscle biopsy samples (Watt et al., 1991). Seminal studies using these intravenous infusions have clearly established that dietary (essential) amino acid ingestion and resistance exercise are potent for the stimulation of muscle protein synthesis, and particularly the myofibrillar protein fraction (Biolo et al., 1995; Borsheim et al., 2002). Although traditional intravenous tracer administration is generally safe provided strict aseptic stable isotope preparation and administration are used (Darmaun & Mauras, 2005), primed constant infusions may be cumbersome when studying exercise effects on muscle protein metabolism given the need to monitor infusion lines during vigorous physical exertion, and/or can add extra time (1.5–2 h) to a participant's research commitment due to the requirement to establish isotope steady‐state. Therefore, the establishment of alternate methodologies that are independent of intravenous tracer administration could provide added flexibility to the study of human muscle protein metabolism under a range of physiological conditions.

Seminal studies using the flooding dose method (i.e., intravenous bolus administration of labeled and unlabeled amino acid) helped establish the importance of exogenous essential amino acids for the postprandial stimulation of muscle protein synthesis (Smith et al., 1992, 1998). Recent advancements in the production of “intrinsically‐labeled” dietary proteins have subsequently demonstrated that dietary amino acids represent essential precursors for the synthesis of skeletal muscle proteins during the immediate postprandial period (Groen et al., 2015). Moreover, labeled foods have also been used to directly model feeding‐mediating muscle protein synthesis rates independent of intravenous tracer infusions in humans (Wall et al., 2013). However, it is often technically and economically challenging to produce these labeled food proteins that are sufficiently enriched to be traced into skeletal muscle proteins, and these difficulties scale with the size of the animal (e.g., chickens vs. lactating cows) and tracer delivery mode (e.g., oral vs. intravenous) for labeled food production (Burd et al., 2013; Pennings et al., 2011; van Vliet et al., 2016). Alternatively, oral ingestion of deuterium oxide (D_2_O) has been used to measure the acute (i.e., 4 h) MyoPS response to essential amino acid ingestion and showed similar postprandial rates as primed‐constant L‐[*ring*‐^13^C_6_]phenylalanine infusion, although these methods have low limits of agreement (Wilkinson et al., 2015).

Therefore, the present proof‐of‐principle study was designed to test the efficacy of a crystalline amino acid beverage labeled with two commonly used tracers of L‐[1‐^13^C]leucine and L‐[*ring*‐^2^H_5_]phenylalanine, each which are differently metabolized in skeletal muscle (Smith et al., 2007), to estimate myofibrillar protein synthesis rates (MyoPS; expressed as FSR) across a range of anabolic stimuli (i.e., fasting, feeding, and feeding and resistance exercise). Particularly leucine has a higher abundance in muscle protein and a more complex metabolic fate (directly oxidized in muscle and is a primary anabolic signaling molecule) when compared to phenylalanine, which is not oxidized in skeletal muscle. We hypothesized that, due to the sensitizing effects of resistance exercise on myofibrillar proteins (Moore, Tang, et al., 2009; Witard et al., 2014), dietary amino acid incorporation and MyoPS would be greatest after feeding and exercise when compared to feeding and tracer‐only ingestion at rest. Furthermore, we hypothesized that despite differences in the relative rates of MyoPS between tracers, the similar digestion/absorption patterns of crystalline amino acids would result in a positive correlation between leucine‐ and phenylalanine‐derived MyoPS as suggested previously with primed constant infusion methods (Harber et al., 2011).

## 2 MATERIALS AND METHODS

### 2.1 Participants

Twenty‐two healthy males (characteristics below) were included in the study and were the same cohorts as previously published (Mazzulla, 2022; Mazzulla et al., 2022). Participants underwent two separate metabolic trials; (i) for whole body protein metabolism (previously published), and; (ii) for determination of MyoPS by oral tracers (present study). Participants were recruited via postings at the University of Toronto and were recreationally active (e.g., performed weightlifting, running, and team‐sport activity) 2–5 times per week for at least 6 months before enrolment. Participants were tracer naïve with no past history in stable isotope amino acid tracer experiments. Participants were considered healthy based on responses to the PAR‐Q+ and a medical history form. All participants were informed of the study purpose, experimental procedures, and potential risks, and written informed consent was obtained from individuals before study participation. The study was performed in accordance with the Declaration of Helsinki and the study protocol was approved by the University of Toronto Research Ethics Board (No. 36752). The study was registered as a Clinical Trial at ClinicalTrials.gov (No. NCT04887727).

### 2.2 Experimental design

A group design was used in the present study. Participants reported to the laboratory for baseline testing 5–7 days before the metabolic trial, which included body composition testing and exercise familiarization. Fat‐free mass was measured by air displacement plethysmography (BodPod, Cosmed USA Inc., Concord, CA) after avoiding food, water, and exercise for at least 4 h before testing. After body composition testing, participants were familiarized with the whole‐body resistance exercise protocol and underwent one‐repetition maximum (1‐RM) testing for the following exercises: (i) dumbbell chest press; (ii) dumbbell bent over row; (iii) leg press; and (iv) leg extension. Participants warmed up with ~50% of their estimated 1‐RM, and weight was progressively added to determine their 1‐RM within 4–5 attempts. After baseline testing, participants were randomly assigned to a rested‐fasted (FAST: *n* = 7, 22 ± 6 years; 72 ± 13 kg body mass; 14 ± 4% body fat), rested‐fed (FED: *n* = 7; 23 ± 5 years; 77 ± 4 kg body mass; 14 ± 4% body fat), or exercise‐fed (EXFED: *n* = 8; 22 ± 2 years; 78 ± 10 kg body mass; 13 ± 5% body fat) condition. Participants were instructed to refrain from alcohol and caffeine consumption as well as vigorous exercise for 48 h before the metabolic trial, and to consume their typical diet. Participants were instructed to record their diet for 48 h before the metabolic trial using a food diary. Dietary intake was analyzed for energy and macronutrient content using The Food Processor® Nutrition Analysis Software (ESHA, Salem, OR).

### 2.3 Metabolic trial

**FIGURE 1:**
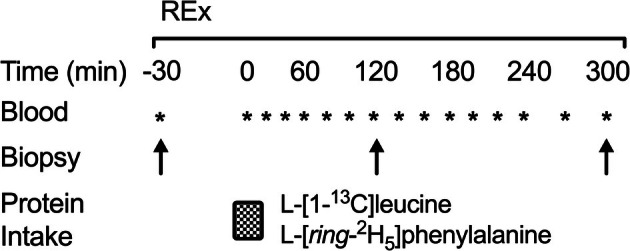
Metabolic trial schematic. REx: Whole‐body resistance exercise performed in EXFED.

Participants reported to the laboratory at ~0700 h after an overnight fast for the metabolic trial (Figure 1). Upon arrival participants rested in a supine position and a PTFE catheter (BD, Franklin Lakes, NJ) was inserted into an antecubital vein for repeated blood sampling. A baseline blood sample was collected in an 8 mL EDTA Vacutainer® (BD) and chilled on ice to determine basal leucine concentrations and background tracer enrichments. The catheter remained patent with a 0.9% saline flush after each subsequent blood draw. Shortly thereafter, a baseline muscle biopsy was collected from the middle region of the *vastus lateralis* using a 5 mm Bergström needle modified for manual suction under 2% lidocaine local anesthesia to determine background muscle‐bound tracer enrichments. Muscle samples were freed from visible blood, fat, and connective tissue and rapidly frozen in liquid nitrogen for further analysis. Each subsequent biopsy was obtained from separate incisions (~2–3 cm apart) in alternating legs. Immediately after this biopsy participants in EXFED performed a bout of whole‐body resistance exercise consisting of 4 × 10 repetitions each of dumbbell bench press, dumbbell bent over row, leg press, and leg extension while participants in FAST and FED rested. Each exercise was performed at 75% of their pre‐determined 1‐RM with ~90 s rest between sets. Whole‐body resistance exercise was selected as the exercise stimulus for the measurement of whole‐body amino acid kinetics (not presented here), and to provide ecological validity for exercised individuals included in the study. Immediately after cessation of exercise (*t* = 0 min) participants ingested a beverage (described below) with (FED and EXFED) or without (FAST) additional nutrition (i.e., amino acids and carbohydrate). Muscle biopsies were collected at *t* = 120 and 300 min after beverage ingestion to measure dietary amino acid incorporation and MyoPS from the oral tracers. Blood samples were collected every 20–30 min after beverage ingestion to determine plasma leucine concentrations and precursor pool enrichments. Plasma was separated from whole blood by centrifugation (3000 × *g* for 10 min at 4°C) and stored at −80°C until further analysis.

### 2.4 Beverage characteristics

Participants in FED and EXFED ingested a beverage containing 0.25 g·kg^−1^ protein as crystalline amino acids (Ajinomoto Co. Inc., Raleigh, NC) modeled on the composition of egg protein based on a test beverage used by Kato et al. (2016), and 0.75 g·kg^−1^ carbohydrate (TANG, Kraft Canada Inc., Mississauga, ON) dissolved in 500 mL water. This protein dose was selected as we (Pennings et al., 2011; Wilkinson et al., 2015) have shown that 0.25 g·kg^−1^ protein per meal maximally stimulates MyoPS in young males. To measure dietary amino acid incorporation and MyoPS from oral tracers the leucine content (~20 mg·kg^−1^) of the beverage was enriched to 25% with L‐[1‐^13^C]leucine (CLM‐468‐PK; Cambridge Isotope Laboratories Inc., Montreal, PQ) and phenylalanine content (~14 mg·kg^−1^) was enriched to 30% with L‐[*ring*‐^2^H_5_]phenylalanine (DLM‐1258‐PK; Cambridge Isotope Laboratories Inc., Montreal, PQ). These enrichment levels were similar to what we (van Vliet et al., 2016) and others (Pennings et al., 2011) have obtained with intrinsically labeled proteins and are suitable to measure MyoPS (Burd et al., 2014). Participants in FAST ingested only the tracers (i.e., ~5 mg·kg^−1^ L‐[1‐^13^C]leucine and ~ 4 mg·kg^−1^ L‐[*ring*‐^2^H_5_]phenylalanine) as a pulse dose (Zhang et al., 2002) dissolved in 200 mL water to establish the metabolic fate of the tracers into myofibrillar proteins independent of the stimulatory effect of additional amino acids and resistance exercise on MyoPS.

### 2.5 Plasma analyses

Plasma leucine concentrations were measured by electron‐impact gas chromatography–mass spectrometry (GC‐MS, 7890B GC, 5977A MSD, Agilent Technologies Inc., Santa Clara CA). Plasma (100 μL) was deproteinized in 100% acetonitrile and centrifuged at 3000 × g for 5 min at 4°C. Free amino acids were dried under N_2_ at 50°C and converted to their *t*‐bdmcs derivatives in acetonitrile/MTBSTFA+1%TBDMCS (Sigma‐Aldrich Canada Co., Oakville, ON), and the mixture was heated for 30 min at 100°C before GC‐MS analysis. Total plasma leucine concentrations were determined by integrating amino acid peak areas of *m* + 0 and *m* + 1 leucine in comparison to a known concentration of *m* + 10 (L‐leucine‐d_10_; DLM‐9423‐PK, Cambridge Isotope Laboratories Inc., Montreal, PQ) as an internal standard using MassHunter Workstation (Version B.07.00, Agilent Technologies Inc.). Plasma L‐[*ring*‐^2^H_5_]phenylalanine enrichments were measured by GC‐MS (Agilent Technologies Inc.) according to previously described methods (Hannaian et al., 2020) and determined by selected ion monitoring (SIM) at *m/z* 148 (*m* + 0) and 153 (*m* + 5) for unlabeled and labeled phenylalanine, respectively, using MassHunter Workstation (Agilent Technologies Inc.). Plasma α‐[1‐^13^C]ketoisocaproate ([^13^C]KIC) enrichments were measured by GC‐MS (Agilent Technologies Inc.) as a surrogate for intramuscular leucyl‐transfer RNA labeling (Watt et al., 1991) according to previously described methods (Mazzulla et al., 2017) and determined by SIM at *m/z* 232 (*m* + 0) and 233 (*m* + 1) for unlabeled and labeled KIC, respectively, using MassHunter Workstation (Agilent Technologies Inc.).

### 2.6 Muscle analyses

Myofibrillar protein‐enriched fractions were isolated from ~25 mg wet muscle tissue as previously described (Hannaian et al., 2020). For L‐[1‐^13^C]leucine, amino acids were derivatized as their *N*‐acetyl‐*N*‐propyl esters (Greenhaff et al., 2008) and myofibrillar‐bound protein enrichments of L‐[1‐^13^C]leucine were measured by gas chromatography‐combustion‐isotope ratio mass spectrometry (Delta plus XP, Thermofisher Scientific, Hemel Hemstead, UK) using established techniques (Kumar et al., 2009).

Samples were treated and analyzed for myofibrillar‐bound protein enrichments of L‐[*ring*‐^2^H_5_]phenylalanine by liquid chromatography–tandem mass spectrometry according to previously described techniques (Hannaian et al., 2020). Briefly, samples were injected onto a 3.5 μm 2.1 mm × 150 mm column (XTerra, Waters, Mississauga, ON) on a 1290 liquid chromatography system (Agilent Technologies Inc.) coupled to a mass spectrometer (Q‐Trap 5500, SCIEX, Framingham, MA). Samples were eluted isocratic using a gradient of 0.1% formic acid and 0.1% formic acid/acetonitrile with a total run time of 3 min. Enrichment ratios were determined by dividing the enriched phenylalanine peak area (i.e., L‐[*ring*‐^2^H_5_]phenylalanine) by the internal standard (i.e., natural phenylalanine) peak area. The transitions monitored were *m/z* 166.1 to 131 for phenylalanine and *m/z* 171.1 to 125 for L‐[*ring*‐^2^H_5_]phenylalanine. Data were collected and analyzed using SCIEX Analyst (version 1.7).

### 2.7 Calculations

Changes in myofibrillar‐bound protein enrichments representing the incorporation of dietary leucine and phenylalanine were determined from the atom percent excess (APE) and mole percent excess (MPE) of L‐[1‐^13^C]leucine (ΔLEU) and L‐[*ring*‐^2^H_5_]phenylalanine (ΔPHE), respectively, over the entire postprandial period using the *t* = 0 and 300 min biopsy (Smith et al., 2007). Due to tissue constraints, only ΔPHE was assessed from *t* = 0 to 120 min.

The fractional synthetic rate (FSR) of myofibrillar proteins representing MyoPS was calculated using the standard precursor‐product method (Rennie et al., 1994):

$$\mathrm{FSR}\;\left(\%h^{-1}\right)=∆E_{p}/E_{m}\times 1/t\times 100$$

where ∆E_p_ is the change in myofibrillar protein‐bound enrichment between two biopsy samples, E_m_ is the average weighted enrichment of the plasma precursor pool across two biopsy samples (calculated using trapezoidal area under the curve divided by change in time), and *t* is the time between biopsies. Plasma [^13^C]KIC and L‐[*ring*‐^2^H_5_]phenylalanine enrichments were used as the precursor pools for leucine (LEUMyoPS) and phenylalanine (PHEMyoPS) derived MyoPS, respectively, due to metabolic and isotopic non‐steady state conditions in the present study, and because the potentially changing intracellular enrichments in response to nonsteady state conditions could not be accurately modeled using only two biopsies (Smith et al., 1992). Due to tissue constraints only phenylalanine derived MyoPS was assessed from *t* = 0 to 120 min.

### 2.8 Statistical analyses

The present study was powered off previous work utilizing a similar group design to compare the effects of feeding and resistance exercise on MyoPS over a 300‐min postprandial period (Moore, Tang, et al., 2009). Specifically, MyoPS was ~43% greater in EXFED (0.070 ± 0.008%·h^−1^) compared to FED (0.049 ± 0.008%·h^−1^), with an effect size of ~2.7 and significant differences between conditions. Therefore, with an α = 0.05 and a β = 0.8, we reasoned that a similar sample size (*n* = 7 per group) would be sufficient to detect a significant difference between FED and EXFED (i.e., our two anabolic conditions). Statistical analyses were performed on SPSS Statistics (Version 26, IBM, Armonk, NY). Differences in time course variables were tested using a mixed‐design two‐factor (time×condition) ANOVA with repeated measures on time. Where sphericity was violated a Greenhouse–Geisser correction was applied to all main effects and interactions, and if data were not normally distributed logarithmic transformations were conducted. Where significant interactions were identified in the ANOVA a Bonferroni post hoc test was performed to determine differences between means for all significant main effects and interactions. A one‐way ANOVA was used to test differences in mean plasma precursor pool enrichments, ΔLEU, ΔPHE, LEUMyoPS, and PHEMyoPS. Where significance was identified in the ANOVA a Bonferroni post hoc test with multiple comparisons was used to identify differences between conditions. Simple linear regression was applied to test for correlations between LEUMyoPS and PHEMyoPS over the 300‐min postprandial period. For all analyses the level of significance was *p* < 0.05, and all results are presented as means ± SD.

## 3 RESULTS

### 3.1 Plasma leucine concentrations

**FIGURE 2:**
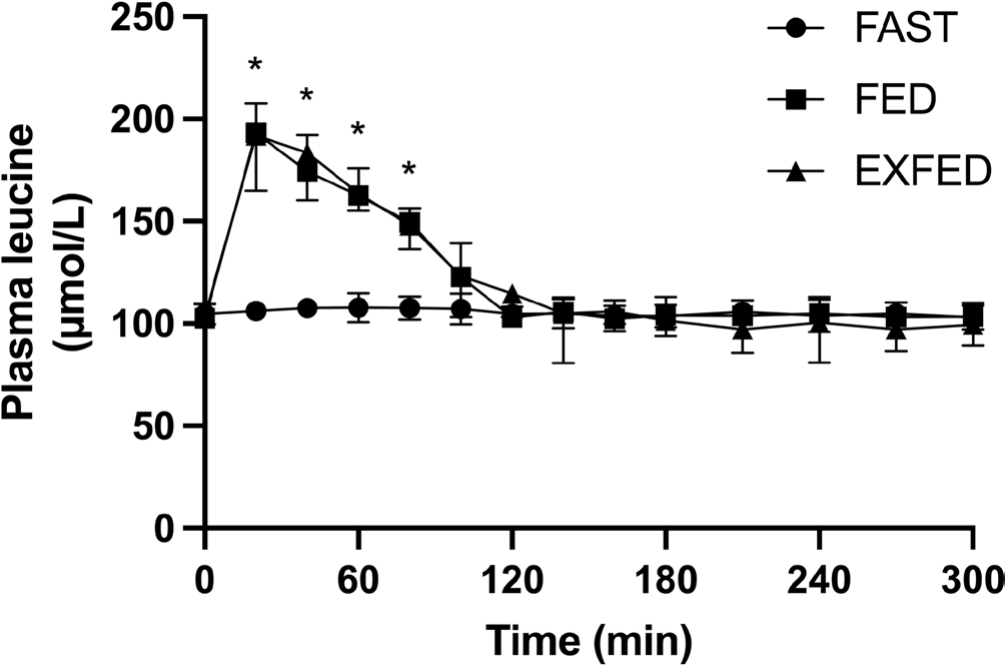
Plasma leucine concentrations (μmol·l^−1^): Time effect, condition effect, and time×condition interaction, all *p* < 0.0001. *Denotes FED and EXFED different from FAST at same time point (all, *p ≤* 0.05). Data are means ± SD. FAST: *n* = 7; FED: *n* = 7; EXFED: *n* = 8. Figure adapted from (Mazzulla, 2022).

There was a time by condition interaction (*p* < 0.0001) for plasma leucine concentrations (Figure 2). Plasma leucine concentrations were greater (all, *p* < 0.0001) in FED and EXFED compared to FAST at *t* = 20, 40, 60, and 80 min with no differences (all, *p* ≥ 0.08) between FED and EXFED at any time point. FED and EXFED plasma leucine concentrations were elevated (all, *p* ≤ 0.01) above basal levels after beverage ingestion and remained elevated until *t* = 80 min, whereas in FAST plasma leucine concentrations did not change (all, *p* > 0.99) from basal values.

### 3.2 Oral leucine

**FIGURE 3:**
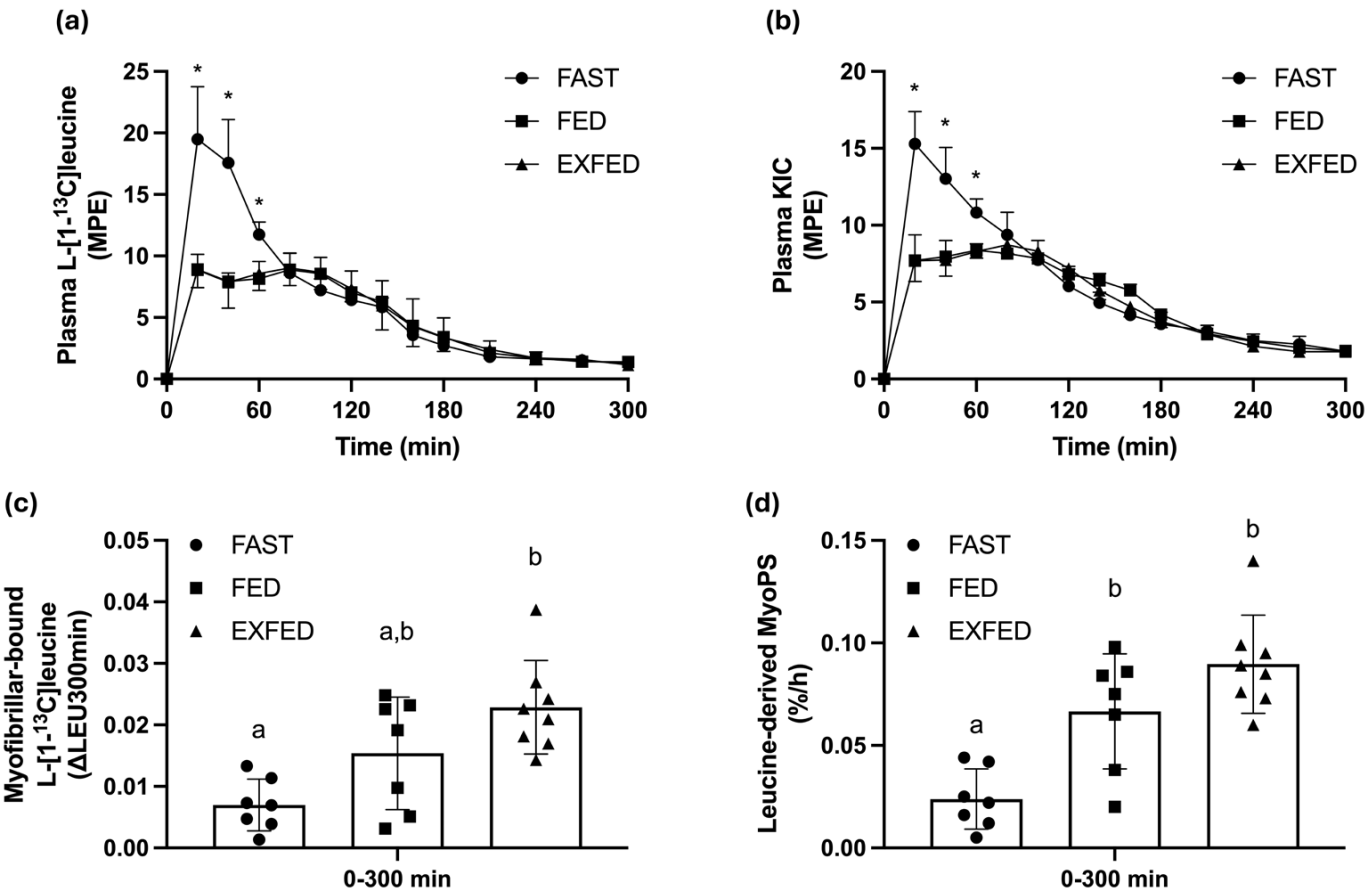
(a) Plasma L‐[1‐^13^C]leucine enrichments (Mole Percent Excess; MPE): Time effect, condition effect, and time×condition interaction, all *p* ≤ 0.0002. *Denotes FED and EXFED different from FAST at same time point (all, *p* ≤ 0.001); (b) Plasma [^13^C]KIC enrichments (MPE): Time effect, condition effect, and time×condition interaction, all *p* < 0.0001. *Denotes FED and EXFED different from FAST at same time point (all, *p* < 0.0001); (c) Change in myofibrillar‐bound L‐[1‐^13^C]leucine enrichments over 300 min (ΔLEU; Atom Percent Excess): Main effect, *p* = 0.002; (d) Leucine derived MyoPS over 300 min (%·h^−1^; LEUMyoPS): Main effect, *p* < 0.0001. All data are means ± SD. FAST: *n* = 7; FED: *n* = 7; EXFED: *n* = 8. Figure adapted from (Mazzulla, 2022).

Individual plasma enrichment curves for all metabolites can be found in Figure S1. There was a time by condition interaction (*p* < 0.0001) for plasma L‐[1‐^13^C]leucine enrichments (Figure 3a). Plasma L‐[1‐^13^C]leucine enrichments were greater (all, *p* ≤ 0.001) in FAST compared to FED and EXFED at *t* = 20, 40, and 60 min with no differences (all, *p* ≥ 0.15) between FED and EXFED at any time point. During all three conditions, plasma L‐[1‐^13^C]leucine enrichments were elevated (all, *p* ≤ 0.05) above basal levels after beverage ingestion and remained elevated until *t* = 300 min.

There was a time by condition interaction (*p* < 0.0001) for plasma [^13^C]KIC enrichments (Figure 3b). Plasma [^13^C]KIC enrichments were greater (all, *p* < 0.0001) in FAST compared to FED and EXFED at *t* = 20, 40, and 60 min with no differences (all, *p* ≥ 0.09) between FED and EXFED at any time point. During all three conditions, plasma [^13^C]KIC enrichments were elevated (all, *p* ≤ 0.05) above basal levels after beverage ingestion and remained elevated until *t* = 300 min.

There was a main effect (*p* < 0.0001) for plasma [^13^C]KIC precursor pool enrichments over the 300‐min postprandial period (data not shown). Precursor pool [^13^C]KIC enrichments were greater (both, *p* = 0.0001) in FAST (6.07 ± 0.46 MPE) compared to FED (5.18 ± 0.19 MPE) and EXFED (5.05 ± 0.21 MPE) with no differences (*p* > 0.99) between FED and EXFED.

There was a main effect (*p* = 0.002) for ΔLEU representing dietary leucine incorporation into myofibrillar proteins over 300 min (Figure 3c). ΔLEU over 300 min was greater (*p* = 0.001) in EXFED compared to FAST with no differences between FED and EXFED (*p* = 0.19) or FAST and FED (*p* = 0.13).

There was a main effect (*p* < 0.0001) for LEUMyoPS over 300 min (Figure 3d; Table 1). LEUMyoPS over 300 min was greater (both, *p* ≤ 0.01) in FED and EXFED compared to FAST with no differences (*p* = 0.20) between FED and EXFED.

**TABLE 1:** Myofibrillar protein synthesis (%/h) by oral leucine and phenylalanine tracers.

Tracer	FAST	FED	EXFED
[^13^C]leucine	0.024 ± 0.015^a^	0.067 ± 0.028^b^	0.090 ± 0.024^b^
[D5]phenylalanine	0.056 ± 0.012^a^	0.098 ± 0.020^b^	0.128 ± 0.034^b^

*Note*: Table depicts mean ± SD for MyoPS in the fasted (FAST; *n* = 7), fed (FED; *n* = 7), and exercise‐fed (EXFED; *n* = 8) conditions. Means within a tracer with different letters are significantly different; *p* < 0.05.

### 3.3 Oral phenylalanine

**FIGURE 4:**
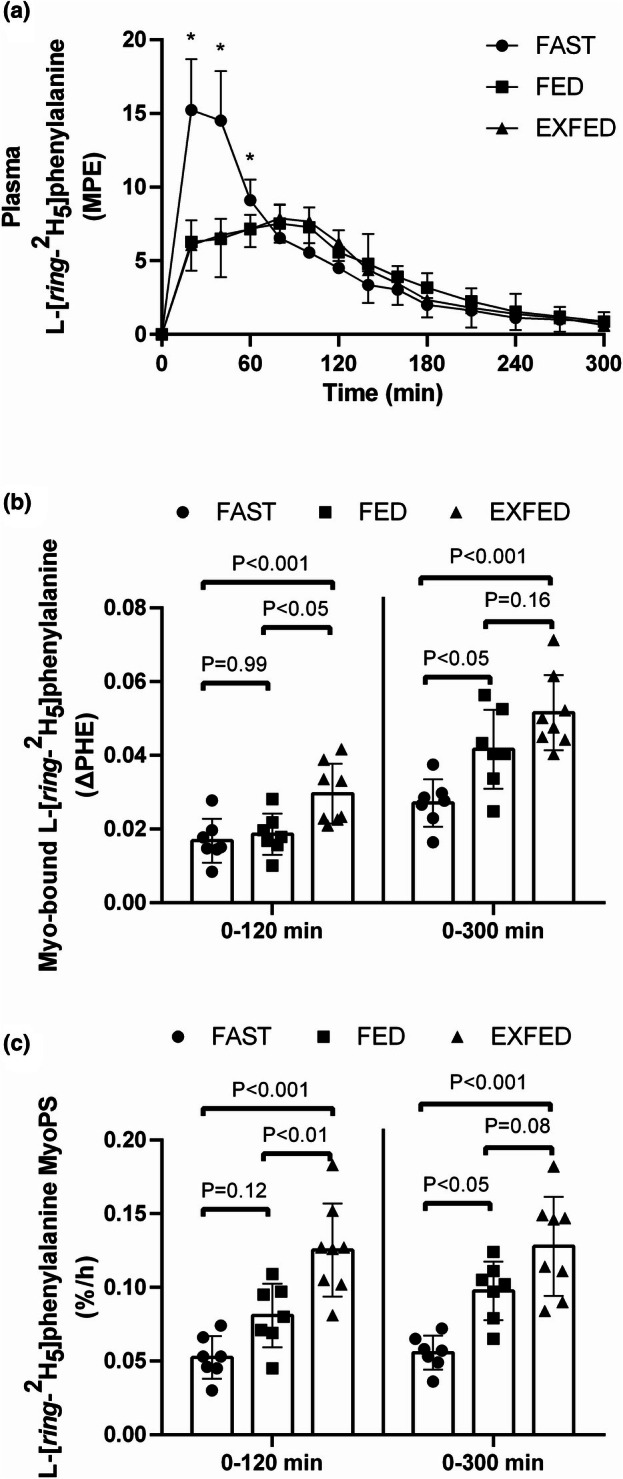
(a) Plasma L‐[*ring*‐^2^H_5_]phenylalanine enrichments (Mole Percent Excess; MPE): Time effect, condition effect, time×condition interaction, all *p* ≤ 0.02. *Denotes FED and EXFED different from FAST at same time point (all, *p* ≤ 0.05); (b) Change in myofibrillar‐bound L‐[*ring*‐^2^H_5_]phenylalanine enrichments over 120 and 300 min (MPE; ΔPHE); ΔPHE over 120 min: Main effect, *p* = 0.003; ΔPHE over 300 min: Main effect, *p* = 0.0003; (c) Phenylalanine derived MyoPS over 120 and 300 min (%·h^−1^; PHEMyoPS); PHEMyoPS over 120 min: Main effect, *p* < 0.0001; PHEMyoPS over 300 min: Main effect, *p* < 0.0001.). All data are means ± SD. FAST: *n* = 7; FED: *n* = 7; EXFED: *n* = 8. Figure adapted from (Mazzulla, 2022).

There was a time by condition interaction (*p* < 0.0001) for plasma L‐[*ring*‐^2^H_5_]phenylalanine enrichments (Figure 4a). Plasma L‐[*ring*‐^2^H_5_]phenylalanine enrichments were greater (all, *p* ≤ 0.05) in FAST compared to FED and EXFED at *t* = 20, 40, and 60 min, with no differences (all, *p* ≥ 0.47) between FED and EXFED at any time point. During all three conditions, plasma L‐[*ring*‐^2^H_5_]phenylalanine enrichments were elevated (all, *p* ≤ 0.05) above basal levels after beverage ingestion and remained elevated until *t* = 180 min.

There was a main effect (*p* = 0.003) for ΔPHE representing dietary phenylalanine incorporation into myofibrillar proteins over 120 min (Figure 4b). ΔPHE over 120 min was greater (both, *p* ≤ 0.02) in EXFED compared to FAST and FED with no differences (*p* = 0.99) between FAST and FED. When assessing plasma L‐[*ring*‐^2^H_5_]phenylalanine precursor pool enrichments over the 120‐min postprandial period, there was a main effect (*p* = 0.0001) for condition whereby they were greater (both, *p* ≤ 0.0005) in FAST (7.92 ± 0.85 MPE) compared to FED (5.76 ± 0.57 MPE) and EXFED (5.96 ± 0.95 MPE) with no differences (*p* > 0.99) between FED and EXFED. This resulted in a main effect (*p* < 0.0001) for PHEMyoPS over 120 min, whereby EXFED was greater (both, *p* ≤ 0.01) compared to FAST and FED with no differences (*p* = 0.12) between FAST and FED (Figure 4c; Table 1).

There was a main effect (*p* = 0.0003) for ΔPHE representing dietary phenylalanine incorporation into myofibrillar proteins over 300 min whereby it was greater (both, *p* ≤ 0.03) in FED and EXFED compared to FAST with no differences (*p* = 0.16) between FED and EXFED (Figure 4b). When assessing plasma L‐[*ring*‐^2^H_5_]phenylalanine precursor pool enrichments over the 300‐min postprandial period there was a main effect (*p* = 0.02) whereby enrichments were greater (both, *p* ≤ 0.05) in FAST (4.89 ± 0.55 MPE) compared to FED (4.15 ± 0.43 MPE) and EXFED (4.06 ± 0.61 MPE) with no differences (*p* > 0.99) between FED and EXFED. This resulted in a main effect (*p* < 0.0001) for PHEMyoPS over 300 min whereby FED and EXFED were greater (both, *p* ≤ 0.02) compared to FAST, with a trend (*p* = 0.08) toward greater PHEMyoPS in EXFED when compared to FED (Figure 4c).

### 3.4 Leucine versus phenylalanine agreement

**FIGURE 5:**
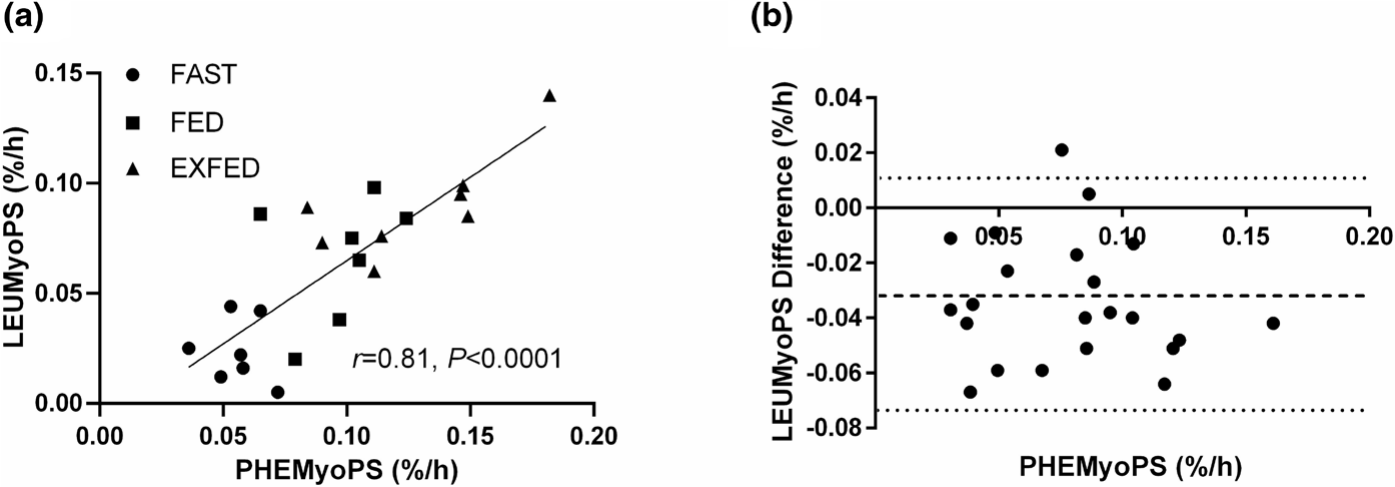
(a) Correlation between leucine (LEUMyoPS) and phenylalanine (PHEMyoPS) derived MyoPS over 300 min: *R* = 0.81, *p* < 0.0001. Total *n* = 22. Line defined by Y = 0.7569X – 0.0108. (b) Bland–Altman Plot illustrating agreement between PHEMyoPS (x‐axis) and LEUMyoPS (bias). Thick hashed line represents mean bias with thin hashed lines the upper and lower 95% confidence interval. Figure adapted from (Mazzulla, 2022).

When collapsed across conditions there was a positive correlation (*r* = 0.81, *p* < 0.0001) between LEUMyoPS and PHEMyoPS over 300 min (Figure 5a). However, assessing the bias revealed a 0.034 ± 0.023%/h bias toward PHEMyoPS rates (Figure 5b).

## 4 DISCUSSION

The present study examined the incorporation of orally delivered L‐[1‐^13^C]leucine and L‐[*ring*‐^2^H_5_]phenylalanine into myofibrillar proteins with tracer alone, feeding and tracer alone, or feeding and tracer combined with resistance exercise in healthy young males. We showed that our complete crystalline amino acid mixture labeled with 25% L‐[1‐^13^C]leucine and 30% L‐[*ring*‐^2^H_5_]phenylalanine detected changes in dietary amino acid incorporation into myofibrillar proteins and the stimulation of MyoPS over a range of anabolic stimuli. Despite a ~31% greater LEUMyoPS and PHEMyoPS observed in EXFED versus FED over 300 min, we were unable to detect statistically significant differences between our two anabolic conditions. A retrospective power calculation based on PHEMyoPS revealed that a sample size of *n* = 13 per group would be required to detect significant differences between FED and EXFED suggesting we may have been underpowered with our between group design, although biopsy timing, as discussed below, may have also contributed to the lack of statistical difference. Nevertheless, the strong positive correlation (*r* = 0.81) we observed between LEUMyoPS and PHEMyoPS indicates that oral single labeled [13C]leucine and multi‐labeled [D5]phenylalanine tracers are qualitatively similar practical tools to detect differences in MyoPS across varying physiological states, especially when intravenous infusions are not possible or the choice depends on available equipment (IRMS vs. LC‐MS/MS).

Participants in FAST ingested an oral dose of L‐[1‐^13^C]leucine and L‐[*ring*‐^2^H_5_]phenylalanine without additional nutrients, resulting in rapid (i.e., within 20 min) peak plasma tracer enrichments without a detectable increase in plasma leucine (and presumably phenylalanine) concentrations. Hyperaminoacidemia of the essential amino acids is required to enhance amino acid availability and incorporation into skeletal muscle (Smith et al., 1992, 1998). Ingesting ~5 mg·kg^−1^ L‐[1‐^13^C]leucine had no detectable effect on plasma leucine concentrations in the present study and, therefore, would presumably not have resulted in any stimulation of muscle protein synthesis rates. However, tracer ingestion alone rapidly labeled the KIC (plasma precursor pool), which is a requirement for measuring muscle protein synthesis rates in the absence of measuring leucyl‐tRNA enrichment and forms the basis for the oral (as compared to intravenous) (Zhang et al., 2002) pulse dose variation used here. The plasma responses to tracer ingestion observed in the present study are similar to Tang and colleagues (Tang et al., 2007) who used an intravenous pulse dose of L‐[*ring*‐^2^H_5_]phenylalanine and observed increased plasma tracer enrichment and in the absence of increases in amino acid concentrations in the fasted state. We recently used this oral L‐[*ring*‐^2^H_5_]phenylalanine tracer approach to measure rates of post‐exercise MyoPS over 2.5 h in the fasted state that were ~2‐fold greater than those in the present study in the absence of changes in amino acid concentration (Hannaian et al., 2020), collectively highlighting it can be used to rapidly (within 2–2.5 h) assess fasted rates of MyoPS in different physiological states. However, our estimated fasted MyoPS rates via oral L‐[1‐^13^C]leucine (~0.024%/h) are slightly lower than those previously reported by the primed constant infusion using plasma KIC enrichment as the precursor (~0.031–0.061%/h) (Smith et al., 2011) but somewhat higher by oral L‐[*ring*‐^2^H_5_]phenylalanine (~0.054%/h) compared to our previous investigations with intravenous administration and plasma enrichment as the precursor (~0.011–031) (Abou Sawan et al., 2018; Paulussen et al., 2023; van Vliet et al., 2019). Therefore, in lieu of pulse intravenous tracer administration (Zhang et al., 2002), our results support oral ingestion as an alternative route of tracer administration that can estimate fasted MyoPS in humans when using the plasma precursor enrichments, although direct comparison to traditional intravenous infusion‐determined rates would require further confirmation.

To determine the ability of oral tracers to detect physiological anabolism, FED and EXFED ingested an oral dose of L‐[1‐^13^C]leucine and L‐[*ring*‐^2^H_5_]phenylalanine with an additional 0.25 g·kg^−1^ crystalline amino acids. This approach could be viewed as a variation of the flooding dose technique that has been used extensively (Garlick et al., 1989; Petersen et al., 2011; Smith et al., 1992, 1998) to measure muscle protein metabolism in response to feeding and/or exercise but is known to stimulate muscle protein synthesis when essential amino acid tracers are utilized. Similar to FAST, we observed rapid increases in plasma tracer enrichments with a concomitant increase in plasma amino acid concentrations, which is in agreement with previous studies utilizing the intravenous flooding technique. However, LEUMyoPS and PHEMyoPS were both substantially increased in FED relative to FAST, highlighting that this oral tracer administration within a “complete protein” (i.e., crystalline amino acids modeled from egg protein) stimulates MyoPS consistent with the known anabolic effects of dietary protein/amino acid ingestion (Churchward‐Venne et al., 2012; Cuthbertson et al., 2005; West et al., 2023). When measured over 300 min, EXFED was numerically (~25%–30%, depending on tracer) greater than FED, which is broadly consistent with the anabolic effect of resistance exercise to sensitize skeletal muscle to dietary amino acids (Churchward‐Venne et al., 2012; Moore, Tang, et al., 2009; Witard et al., 2014). Moreover, the apparently greater MyoPS in EXFED is consistent with our previously reported mTORC1‐related signaling (Hodson et al., 2022) and whole body leucine retention (Mazzulla et al., 2022) in the same cohorts. However, it is notable that PHEMyoPS in EXFED was only statistically greater than FED when measured over 2 h, which suggests consideration of biopsy timing may be important when assessing differences in MyoPS between exercised and rested skeletal muscle. For example, the postprandial stimulation of MyoPS with rapidly digested dietary AA (e.g., crystalline AA or whey protein) may only persist for 90–180 min (Atherton et al., 2010; Churchward‐Venne et al., 2012; Moore, Tang, et al., 2009) whereas resistance exercise can sustain this for up to 300 min (Churchward‐Venne et al., 2012; Moore, Robinson, et al., 2009). Thus, the lack of statistical differences between FED and EXFED may be related to the low plasma tracer enrichment (~1%–2%) that could have limited the differential myofibrillar tracer incorporation over the final hour of our protocol. In contrast, reducing the incorporation time to 4 h may be more suitable for rapidly digested AA‐induced stimulation of MyoPS given this would likely capture the entire postprandial period while still maintaining detectable plasma tracer enrichment in both FED and FAST.

Importantly, we used two different essential amino acid tracers as it has been suggested in rodents that plasma leucine may be primarily directed toward oxidation after its intramuscular uptake whereas intracellular leucine from protein breakdown is preferentially reutilized for protein synthesis (Schneible et al., 1981), although whole‐body research in humans does not support this contention (Boirie et al., 1996). In contrast, phenylalanine has only one fate (protein synthesis) within skeletal muscle (Groen et al., 2015), and may have influenced the choice to use phenylalanine in previous intrinsically labeled protein research (Groen et al., 2015; Pennings et al., 2011; van Loon et al., 2009). Regardless of the tracer utilized, both were able to detect physiological differences in MyoPS between across the range of anabolic stimuli and displayed a strong correlation, suggesting these tracers are equally effective at estimating MyoPS when administered orally. However, rates of MyoPS were not quantitively similar between tracers, which has been noted previously with constant infusion and flooding dose techniques (Smith et al., 1998, 2007). The reason for this discrepancy is not clear within the confines of our study, especially with the apparently paradoxically greater MyoPS determined by phenylalanine as compared to leucine given that plasma KIC enrichment is generally considered a more accurate proxy for intramuscular enrichment (Watt et al., 1991). Nevertheless, the mean difference in MyoPS between FAST and FED (~0.043 vs. ~0.42%/h, respectively) and FED and EXFED (~0.023 vs. ~0.03%/h, respectively) were similar between leucine and phenylalanine tracers, respectively. Thus, consistent with previous conclusions by others (Smith et al., 2007), these oral tracers cannot be used interchangeably within a single study nor be quantitatively compared to each other but can still provide qualitatively similar conclusions on MyoPS across physiological states.

In conclusion, we demonstrated that oral delivery of essential amino acid tracers can detect physiological changes in MyoPS during fasting, feeding at rest, and feeding after resistance exercise. These findings may enhance the feasibility of using free leucine and/or phenylalanine tracers as a tool to safely and quickly detect physiological differences in MyoPS in healthy and clinical populations and/or in situations where intravenous infusion methods may not be readily applied.

## AUTHOR CONTRIBUTIONS

M.M. and D.R.M. conceived and designed research; D.A.K. provided medical oversight; M.M., N.H., P.J.S., K.S., and P.J.A. performed experiments; M.M., N.H., and P.J.S. analyzed data; M.M., N.H., P.J.S., N.A.B., and D.R.M. interpreted results of experiments; M.M. and D.R.M. drafted manuscript with input from N.A.B.; all authors edited, revised, and approved of final version of manuscript.

## FUNDING INFORMATION

This study was supported in part by the University of Toronto Connaught New Researcher Award and a Natural Sciences and Engineering Research Council Discovery Grant (No. RGPIN‐2023‐05382) awarded to D.R.M. M.M. was supported by an Ontario Graduate Scholarship. N.H. was supported by a Mitacs Accelerate Postdoctoral Fellowship (No. IT15730).

## CONFLICT OF INTEREST STATEMENT

No conflicts of interest, financial or otherwise, are declared by the authors.

## ETHICS STATEMENT

The study was performed in accordance with the Declaration of Helsinki and the study protocol was approved by the University of Toronto Research Ethics Board (No. 36752).

## Supporting information


Figure S1.

## Data Availability

The data obtained and analyzed in this study can be obtained upon reasonable request to the corresponding author.
