# Supplementary material for: Oral amino acid tracer delivery detects feeding and exercise changes in myofibrillar protein synthesis rates in male adults
Source: Physiol Rep. 2026 Mar 3;14(5):e70776. doi: 10.14814/phy2.70776 (PMC12956835; doi:10.14814/phy2.70776)
Supplement: Supplementary file 1 — Figure S1. [file PHY2-14-e70776-s001.pdf]

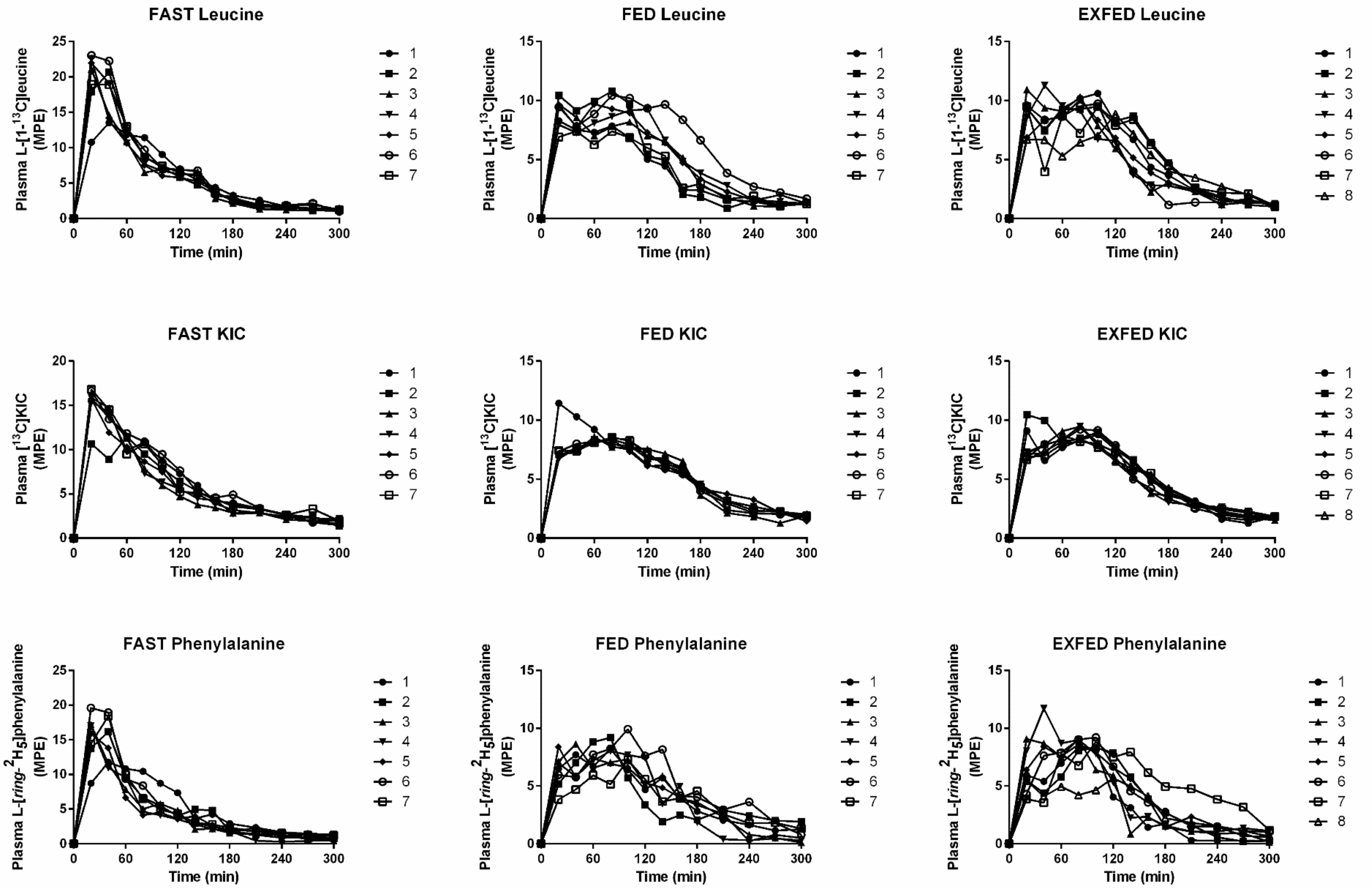

Supplementary figure 1. Individual plasma leucine (top row), keto-isocaproate (KIC), and phenylalanine enrichment curves in mole percent excess (MPE) for fasted (FAST), fed (FED), and exercise fed (EXFED).
